# Supplementary material for: Reconciling Mining with the Conservation of Cave Biodiversity: A Quantitative Baseline to Help Establish Conservation Priorities
Source: PLoS One. 2016 Dec 20;11(12):e0168348. doi: 10.1371/journal.pone.0168348 (PMC5173368; doi:10.1371/journal.pone.0168348)
Supplement: S1 Dataset — (ZIP) [file pone.0168348.s002.zip › Taxa/Serra Norte/SN_2013/N4E/Lista N4E-61.pdf]

CAVIDADE N4E-0061

Classe	Ordem	Fam/Outros	Gên/Outros	Espécie	S-U	U-S	Zon.
Arachnida	Acari	(Oribatida)		sp.8	X	X	P
Arachnida	Acari	Ascidae		sp.1	X		A
Arachnida	Acari	Eupodidae		sp.1	X		P
Arachnida	Acari	Ixodidae	*Amblyomma*	*cajennense*		X	
Arachnida	Acari	Laelapidae		sp.3	X		P
Arachnida	Acari	Mesostigmata		jovem	X	X	P
Arachnida	Acari	Prostigmata		sp.49	X		P
Arachnida	Acari	Trombiculidae		sp.42	X	X	P
Arachnida	Acari	Trombidiidae	Microtrombidiinae	sp.		X	
Arachnida	Amblypygi	Charinidae	*Charinus*	sp.1	1	X	A
Arachnida	Amblypygi	Phrynidae	*Heterophrynus*	*longicornis*	3		
Arachnida	Araneae	Corinnidae		jovem	2	1	P/A
Arachnida	Araneae	Ctenidae		jovem		1	
Arachnida	Araneae	Ochyroceratidae	*Ochyrocera*	sp.1	X	X	P/A
Arachnida	Araneae	Ochyroceratidae	*Speocera*	sp.		X	
Arachnida	Araneae	Oonopidae	aff. *Xyccarph*	sp.1	X		P
Arachnida	Araneae	Pholcidae		jovem	X	X	P/A
Arachnida	Araneae	Theridiosomatidae	*Plato*	sp.1	X	X	P/A
Arachnida	Opiliones	Escadabiidae		sp.2	7	X	P
Arachnida	Opiliones	Stygnidae	*Protimesius*	*gracilis*	1		P
Arachnida	Pseudoscorpiones	Bochicidae		sp.1	X		P
Arachnida	Pseudoscorpiones	Chernetidae	*Spelaeochernes*	sp.1	X	X	P
Arachnida	Pseudoscorpiones	Chthoniidae	*Pseudochthonius*	sp.3	X	X	
Chilopoda	Scolopendromorpha	Scolopocryptopidae	*Newportia*	*ernsti*		X	
Chilopoda	Scutigeromorpha	Pselliodidae		jovem	1		P
Diplopoda	Polydesmida	Chelodesmidae		sp.3	1	X	A
Diplopoda	Polydesmida	Pyrgodesmidae		sp.1	X	X	P/A
Diplopoda	Spirostreptida	Pseudonannolenidae	*Pseudonannolene*	jovem	19	X	P
Entognatha	Collembola	Cyphoderidae		sp.1	X		P/A
Entognatha	Collembola	Entomobryidae		sp.4	X		A
Entognatha	Collembola	Paronellidae		sp.4	X		P
Entognatha	Collembola	Sminthuroidea		sp.		X	
Entognatha	Diplura	Campodeidae		sp.1		X	
Insecta	Blattodea			jovem	1		P
Insecta	Coleoptera	Staphylinidae		sp.		X	
Insecta	Coleoptera	Tenebrionidae		sp.		X	
Insecta	Coleoptera			jovem	X		P/A
Insecta	Diptera	Chironomidae		sp.	X		P
Insecta	Diptera	Drosophilidae	*Drosophila*	sp.		X	
Insecta	Diptera	Phoridae		sp.		X	
Insecta	Diptera	Psychodidae	*Breviscapus*	sp.	X	X	P
Insecta	Diptera	Psychodidae	*Sciopemyia*	*sordellii*	X	X	
Insecta	Diptera	Tipulidae	Tipulinae	sp.	X		P
Insecta	Heteroptera	Cydnidae		sp.	X	X	P/A
Insecta	Homoptera	Cixiidae		jovem	X		P/A
Insecta	Hymenoptera	Bethylidae		sp.		X	
Insecta	Hymenoptera	Diapriidae		sp.1	X		A
Insecta	Hymenoptera	Formicidae	*Apterostigma*	sp.		X	
Insecta	Hymenoptera	Formicidae	*Neivamyrmex*	sp.1	X		P/A

Insecta	Hymenoptera	Formicidae	*Pachycondyla*	sp.2	X		P/A
Insecta	Hymenoptera	Formicidae	*Scelionidae*	sp.1		X	
Insecta	Hymenoptera	Formicidae	*Solenopsis*	sp.1	X	X	P/A
Insecta	Isoptera	Termitidae	*Nasutitermes*	sp.	X		A
Insecta	Lepidoptera	Noctuoidea		sp.5	4		P
Insecta	Lepidoptera	Tineoidea		jovem		X	
Insecta	Orthoptera	Phalangopsidae	*Paraclodes*	sp.	1		
Insecta	Orthoptera	Phalangopsidae	*Phalangopsis*	sp.	230	X	
Insecta	Psocoptera	Epipsocidae		jovem	X		P
Insecta	Thysanura	Nicoletiidae		jovem	X		P
Gastropoda	Pulmonata	Bulimulidae	*Naesiotus*	sp.	X		P
Gastropoda	Pulmonata	Subulinidae	*Lamellaxis*	sp.		X	
Gastropoda	Pulmonata	Systrophiidae	*Happia*	sp.	X		P
Malacostraca	Isopoda	Armadillidae		sp.1	X		P
Malacostraca	Isopoda	Philosciidae		sp.1	X	X	P/A
Malacostraca	Isopoda	Scleropactidae	*Circoniscus*	*buckupi*		X	
Amphibia	Anura	Ranidae		sp.		1	P
Amphibia	Anura	Strabomantidae	*Pristimantis*	cf. *fenestratus*		15	P/A
Mammalia	Chiroptera	Phyllostomidae	*Carollia*	*perspicillata*	24		P/A
Mammalia	Chiroptera	Phyllostomidae	*Micronycteris*	*microtis*	2		P/A
Mammalia	Chiroptera	Phyllostomidae		sp.		20	P/A
Mammalia	Rodentia	Cricetidae	*Rhipidomys*	sp.	3		A
